# Inhibition of proteinase-activated receptor 2 (PAR2) decreased the malignant progression of lung cancer cells and increased the sensitivity to chemotherapy

**DOI:** 10.1007/s00280-023-04630-8

**Published:** 2024-01-04

**Authors:** Hongjie Huo, Yu Feng, Qiong Tang

**Affiliations:** Department of Respiration Medicine, Tianjin Union Medical Center, Tianjin, 300121 China

**Keywords:** Lung cancer, PAR2, Melittin, Gefitinib, Drug resistance

## Abstract

**Objectives:**

This study aimed to study the effect of protease-activated receptor 2 (PAR2) on the proliferation, invasion, and clone formation of lung cancer cells. It also aimed to evaluate the inhibitory effect of melittin on PAR2 and the anti-lung cancer effect of melittin combined with gefitinib.

**Methods:**

The correlation between the co-expression of PAR2 and epithelial–mesenchymal transition (EMT) markers was analyzed. PAR2 in A549 and NCI-H1299 cells was knocked down using siRNA. MTT assay, Transwell assay, and colony formation assay were used to detect the effects of PAR2 on cell proliferation, invasion, and clone formation. The anti-cancer effect of PAR2 knockdown on gefitinib treatment was analyzed. The synergistic effect of melittin on gefitinib treatment by inhibiting PAR2 and the underlying molecular mechanism were further analyzed and tested.

**Results:**

The expression of PAR2 was upregulated in lung cancer, which was associated with the poor prognosis of lung cancer. PAR2 knockdown inhibited the stemness and EMT of lung cancer cells. It also inhibited the proliferation, invasion, and colony formation of A549 and NCI-H1299 cells. Moreover, PAR2 knockdown increased the chemotherapeutic sensitivity of gefitinib in lung cancer. Melittin inhibited PAR2 and the malignant progression of lung cancer cells. Melittin increased the chemotherapeutic sensitivity of gefitinib in lung cancer by inhibiting PAR2.

**Conclusion:**

PAR2 may promote the proliferation, invasion, and colony formation of lung cancer cells by promoting EMT. Patients with a high expression of PAR2 have a poor prognosis. Inhibition of PAR2 increased the chemotherapeutic sensitivity of gefitinib. PAR2 may be a potential therapeutic target and diagnostic marker for lung cancer.

**Supplementary Information:**

The online version contains supplementary material available at 10.1007/s00280-023-04630-8.

## Introduction

Lung cancer is a malignant tumor with the highest morbidity and mortality in the world [1, 2]. At least the two main types of primary lung cancer, small cell lung cancer (SCLC) and non-small cell lung cancer (NSCLC) must be mentioned, with about NSCLC comprising 80–85% of lung cancers. Moreover, NSCLC are subgrouped into adenocarcinoma, squamous cell carcinoma, and large cell carcinoma. Given the insidious symptoms of this disease, most patients with lung cancer are already in the advanced stage when they visit the doctor, resulting in poor prognosis [3]. Even after surgical treatment, the tumor is prone to recurrence and migration, leading to treatment failure and short survival time [1]. With the deepening of the research on tumor pathogenesis, molecular targeted therapy has become a hot spot, such as tyrosine kinase inhibitors (TKIs) of epidermal growth factor receptor (EGFR) [4]. Anaplastic lymphoma kinase fusion mutation is also a new molecular target for the treatment of non-small cell lung cancer (NSCLC) [5]. However, some patients develop drug resistance and are unsuitable for molecular targeted therapy. The emergence of drug resistance greatly affects the use of targeted drugs [6–8]. How to overcome the drug resistance and elucidate the underlying mechanism is the key problem.

Protease-activated receptors (PARs) can be expressed in some tumor cells [9, 10]. PARs have been closely related to tumor growth and metastasis [11]. PAR2 has been associated with tumor metastasis [12]. After PAR2 is activated, it may transcriptionally activate a certain cytokine or change its expression. Activation of PARs causes signal transduction, leading to cell proliferation and migration [13]. In MDA-MB-231 cells, the TF-FII A complex specifically upregulates the expression of IL-8 through PAR2, resulting in significantly enhanced cell migration [14]. MMPs induce the production of transforming growth factor-α (TGF-α), which mediates the regulation of the activation of EGFR and the continuous phosphorylation of downstream MAPK, leading to cell proliferation and migration [15]. However, the role of PAR2 in lung cancer, especially in lung cancer EMT and chemotherapeutic sensitivity, remains to be further studied.

Reversible EGFR-TKIs (gefitinib) are important targeted agents for the treatment of NSCLC [16]. However, patients with primary drug-resistant EGFR-TKIS lung cancer cannot benefit from the treatment, leading to the continuous search for new treatment methods [17]. Melittin is a small protein extracted from bee venom [18]. Melittin has antibacterial, antiviral, anti-inflammatory, and analgesic effects [19]. Melittin exerts inhibitory effects on various tumor cells, and its targets are diverse, including direct killing of cells, inhibition of cell proliferation, promotion of cell apoptosis, and inhibition of cell angiogenesis [20–22].

In this study, we investigated the effects of melittin on the proliferation and apoptosis of NSCLC cells. The molecular mechanism by which melittin enhances the effect of gefinitib through PAR2 inhibition was also discussed. This study aims to find a safe and effective method for the clinical treatment of NSCLC.

## Methods

### Cell culture

The Normal human lung epithelial cells BEAS-2B and lung cancer cell lines H460, A549 and NCI-H1299 were purchased from American Type Culture Collection (ATCC, Manassas, VA, USA) and cultured in RPMI 1640 supplemented with 10% fetal bovine serum (FBS). The culture was placed in an incubator at 37 ℃ and 5% CO_2_. When the bottom of the culture flask was 70–80% full of cells, digestion and passage were performed. Cells in the logarithmic growth phase were used for subsequent experiments.

### Cell transfection

Firstly, the siRNA and transfection reagent were prepared. The siRNA used was specifically designed for targeted silencing of PAR2. The sequences for the siRNA non-targeting control were as follows: forward: 5’-GCGACGAUCUGCCUAAGAUdTdT-3’, reverse: 5’-AUCUUAGGCAGAUCGUCGCdTdT-3’. For the siRNA targeting PAR2: si-PAR2#1: the sequence was 5ʹ-AAUGGCAUGGCCCUCUGGAUC (dTdT)-3ʹ. si-PAR2#2: the sequence was 5’-GCUCUGCAAGGUGCUCAUUGGCUUU-3’. For the transfection process, 100 μL of Opti-MEM was used as a medium. To this, the siRNA and transfection reagent were added. The mixture was gently pipetted to ensure a homogeneous distribution of the siRNA and transfection reagent within the Opti-MEM. Once the siRNA and transfection reagent were thoroughly mixed into the Opti-MEM, the mixture was allowed to stand for 10 min. This incubation period is crucial as it allows the formation of siRNA-lipid complexes, which are necessary for efficient transfection. Following the 10-min incubation, the siRNA-transfection reagent mixture was added dropwise to a 6-well plate containing the cells. Care was taken to distribute the drops evenly across the well to ensure uniform transfection. The cells were then cultured in a 10% FBS medium. The culture was placed in an incubator set at 37 ℃ and 5% CO_2_. These conditions were maintained to ensure optimal cell growth and siRNA uptake. The cells were monitored regularly to assess the transfection efficiency and to ensure that the cells were not undergoing any adverse effects due to the transfection process. Since the transfection efficiency of the two siRNA was different, and si-PAR2#2 could not completely silence the expression level of PAR2 effectively, we selected si-PAR2#1 with high knockout efficiency for the subsequent experiment (Figure S1).

### CancerSEA website analysis

CancerSEA is a multi-functional website aimed at comprehensively exploring the different functional states of cancer cells at the single-cell level [7, 23]. In this study, CancerSEA was used to investigate the correlation between different functional states of the PAR2 gene and lung cancer. An average association was observed between PAR2 and different functional states of lung cancer, including invasion, metastasis, proliferation, and EMT.

### Human protein atlas analysis

In this study, the Human Protein Atlas [24, 25] was used to analyze the expression level of PAR2 in normal lung tissues and lung cancer tissues. The correlation between different expression levels of PAR2 and prognosis of patients with lung cancer was analyzed.

### qRT-PCR

Total RNA was extracted using the RNA extraction kit in accordance with the manufacturer’s instructions. It was then reverse-transcribed into cDNA using a reverse transcription kit. Fluorescence quantitative PCR was performed using Roche’s SYBR Green Master Mix. The Q-PCR reaction system was as follows (20 μL): 10 μL of SYBR qPCR Mix, 0.8 μL each of qPCR upstream and downstream primers (10 μmol/L), 2 μL of cDNA, 0.4 μL of 50 × Rox reference dye, and de-RNase water until 20 μL. Primers were synthesized by Sangon Biotech Co., Ltd. The sequences are displayed in Table 1. The reaction system on the machine detection was as follows: pre-denaturation at 95 ℃ for 1 min, followed by 95 ℃ for 30 s, 60 ℃ for 40 s, 40 cycles. All samples were tested three times with three parallel holes. Gene transcription level was corrected by GAPDH by using the 2^−ΔΔCT^ method.

**Table 1:** The sequences of the primers used in qRT-PCR

Genes	Forward (5’-3’)	Reverse (5’-3’)
PAR2	TTTCTCTCGGTGCGTCCAG	GTTCCTTGGATGGTGCCACT
Oct4	CTTGCTGCAGAAGTGGGTGGAGGAA	CTGCAGTGTGGGTTTCGGGCA
SOX2	CCCTGTGGTTACCTCTTCCTC	CGCTCTGGTAGTGCTGGGAC
Nanog	AATACCTCAGCCTCCAGCAGATG	TGCGTCACACCATTGCTATTCTTC
Snail1	CCTCCCTGTCAGATGAGGAC	CCAGGCTGAGGTATTCCTTG
Slug	GGGGAGAAGCCTTTTTCTTG	TCCTCATGTTTGTGCAGGAG
Twist1	GCAAGAAGTCGAGCGAAGAT	GCTCTGCAGCTCCTCGAA
E-cadherin	TGCCCAGAAAATGAAAAAGG	GTGTATGTGGCAATGCGTTC
Vimentin	GAGAACTTTGCCGTTGAAGC	GCTTCCTGTAGGTGGCAATC
GAPDH	GGAGCGAGATCCCTCCAAAAT	GGCTGTTGTCATACTTCTCATGG

### Cell proliferation was detected by MTT

Cells in the logarithmic growth phase were collected. Cells at a density of 3 × 103 cells per well were inoculated into 96-well plates. After culture for 12 h, the supernatant was discarded. Melittin (1, 2, 4 mg/L) and 200 μL of gefitinib medium (1 μmol/L) were added to each well CCCCC [26–28]. Then, 20 μL of MTT (5 mg/mL) was added to each well after 48 h in the incubator. The cells were cultured at 37 ℃ for 4 h and then added with 150 μL of DMSO. Before detection, the cells were shaken and mixed gently, and the enzyme-linked detector was used to detect the optical density value of each hole at the wavelength of 490 nm (D(450)). The cell proliferation inhibition rate was calculated according to the following formula: Inhibition rate of cell proliferation = ((optical density value of control group-optical density value of drug treatment group)/ optical density value of control group) × 100. The experiment was repeated three times.

### Transwell experiment

Matrigel (serum-free medium) was diluted to the desired concentration by adding 30 μL of each Transwell chamber and allowed to set. Serum-free 1640 medium was used to adjust the cell concentration to 2 × 105 cells/mL. The upper and lower compartments of the Transwell chamber were added with 200 μL of cell suspension and 600 μL of RPMI 1640 containing 10% serum, respectively. The Transwell chambers were incubated for 48 h. Unmigrated cells on the membrane were gently wiped off using a cotton swab. The filter membrane was fixed with methanol for 30 min. The cells were stained with 0.1% crystal violet solution for 20 min. The number of cells crossing the membrane was counted and compared under a microscope. The total number of cells was counted in five fields of vision, namely, upper, lower, left, right, and middle, and averaged.

### Clone formation experiment

Cells in the logarithmic growth phase were inoculated into a 6-well plate at a cell density of 2 × 103 cells/well. After 24 h of culture, RPMI 1640 medium containing 1% FBS was replaced. Then, melittin or gefitinib was added in different concentrations. After 3 days of drug intervention, RPMI 1640 medium containing 10% FBS was replaced for further culture for 10 days. At the end of the experiment, the cells were washed once with phosphate-buffered saline. The samples were fixed with 0.5% crystal violet (prepared with 10% methanol) and stained for 15 min. The number of clones formed by the cells was recorded.

### Western blot

Cells in each group were lysed with RIPA lysate (1 mL RIPA + 10μL PMSF + 10μL aprotinin) for 30 min and operated on ice. Protein concentration was measured by BCA method, and loading buffer was added to denatured protein. Prepare 10% SDS-PAGE and add 20 μg protein sample to each well. Use wet transfer to transfer to PVDF membrane. 5% skimmed milk powder was closed for 2 h. Primary antibody of PAR2 (ab180953, Abcam, Cambridge, MA, USA), Oct4 (ab200834), SOX2 (ab171380), Nanog (ab109250), Snail1 (ab31787), Slug (ab51772)), Twist1 (ab50887)), E-cadherin (ab40772), Vimentin (ab92547) was diluted at 1: 1000 TBST at 4 ℃ overnight. The 1: 5000 diluted secondary antibody was added and incubated for 2 h at room temperature. ECL luminescence kit was developed and analyzed after fixing.

### Statistical analysis

Statistical analysis was performed using SPSS 17.0 statistical software. Measurement data were expressed as mean ± standard deviation. Student’s t-test was used for comparison between the two groups. One-way ANOVA followed by Tukey’s post-hoc test was used for multi-component comparison. Statistical significance was considered at P < 0.05.

## Results

### PAR2 expression is upregulated in lung cancer, which is associated with poor prognosis of lung cancer

**Fig. 1:**
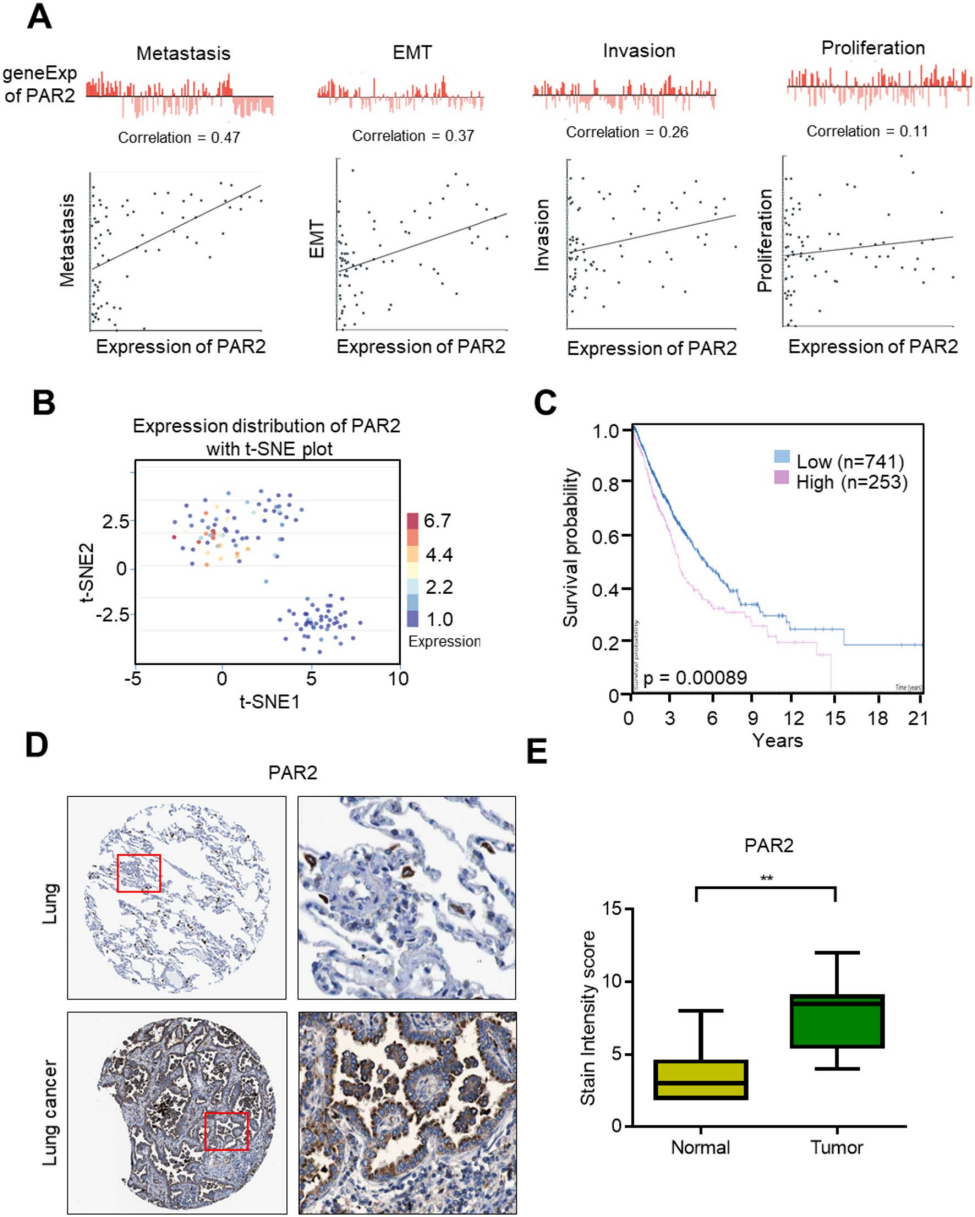
PAR2 is up-regulated in lung cancer and is associated with poor prognosis of lung cancer. **A** Analyze the correlation between PAR2 expression and different functions of lung cancer on the CancerSEA website. **B** t-SEN analysis of cells with different PAR2 expression levels. **C** Survival analysis of lung cancer patients with different PAR2 expression levels. **D** PAR2 immunohistochemical test results in normal lung tissue and lung cancer tissue. The experimental results were obtained from The Human Protein Atlas website. **E** The statistical results of immunohistochemical detection of PAR2 in normal lung tissue and lung cancer tissue. **, P < 0.01. Data are represented as the means ± SD

Single-cell sequencing analysis can solve tumor heterogeneity, improve the accuracy of experimental data analysis, and provide valuable help for the diagnosis, detection, and treatment of diseases. This study first analyzed the correlation between PAR2 and functional properties of lung cancer through the CancerSEA website. Correlation analysis results of PAR2 in multiple functional states of lung cancer showed that PAR2 expression was positively correlated with the metastasis, EMT, invasion, and proliferation of cells (Fig. 1A). The developmental process that PAR2 may participate in was analyzed. The scatter plot of T-SEN functional analysis describes the distribution of cells, with each dot representing a cell and the color of the dot representing the expression of the gene in the cell. T-SEN analysis showed that the low expression of PAR2 in lung cancer cells clumped together. At the same time, the highly PAR2-expressing cell population clumped together (Fig. 1B). Survival analysis showed that PAR2 expression was associated with a poor prognosis of lung cancer. Patients with high PAR2 expression had a short survival time (Fig. 1C). These results suggest that high expression of PAR2 in patients with lung cancer indicates a poor prognosis. Thus, it may be used as a prognostic indicator in patients with lung cancer. Furthermore, the differences in the expression levels of PAR2 in lung tissues and lung cancer tissues were analyzed through The Human Protein Atlas website. Immunohistochemical test results showed that the expression of PAR2 was significantly higher in lung cancer tissue than in normal lung tissue (Figs. 1D, E).

### PAR2 was associated with EMT markers

**Fig. 2:**
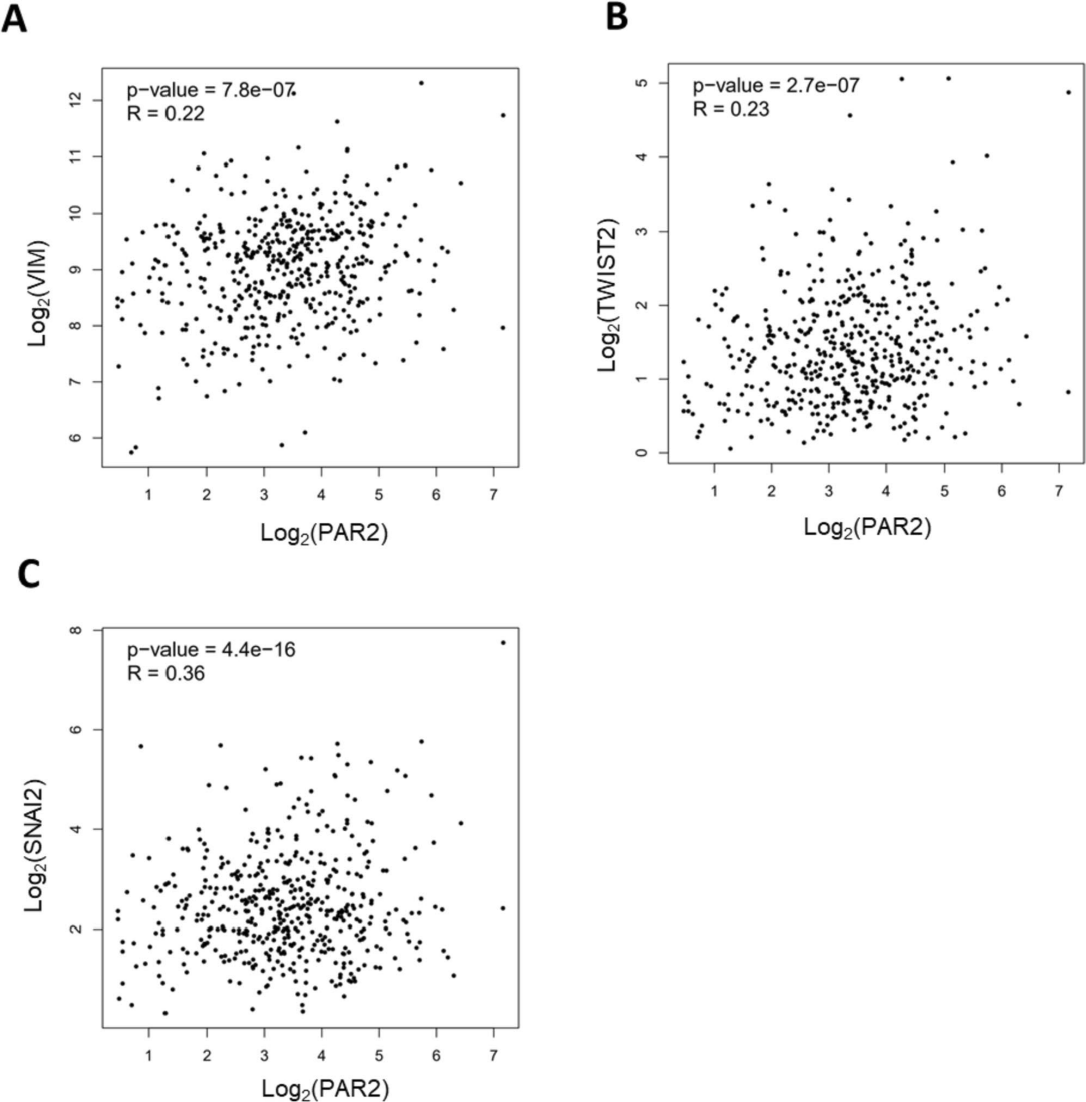
PAR2 was associated with EMT markers. **A** PAR2 expression is positively correlated with Vimentin co-expression. **B** The expression of PAR2 is positively correlated with the co-expression of TWIST2. **C** PAR2 expression is positively correlated with SNAI2 co-expression

Bioinformatics data analysis revealed that PAR2 plays a role in lung cancer. We further analyzed the co-expression correlation between PAR2 and EMT genes. As shown in Fig. 2A, PAR2 was co-positively correlated with the mesenchymal marker vimentin. Further detection revealed that the expression level of PAR2 was co-positively correlated with transcription factors Twist1 and SNAI2 (Figs. 2B, C).

### Knock down PAR2 to inhibit lung cancer cell stemness and EMT

**Fig. 3:**
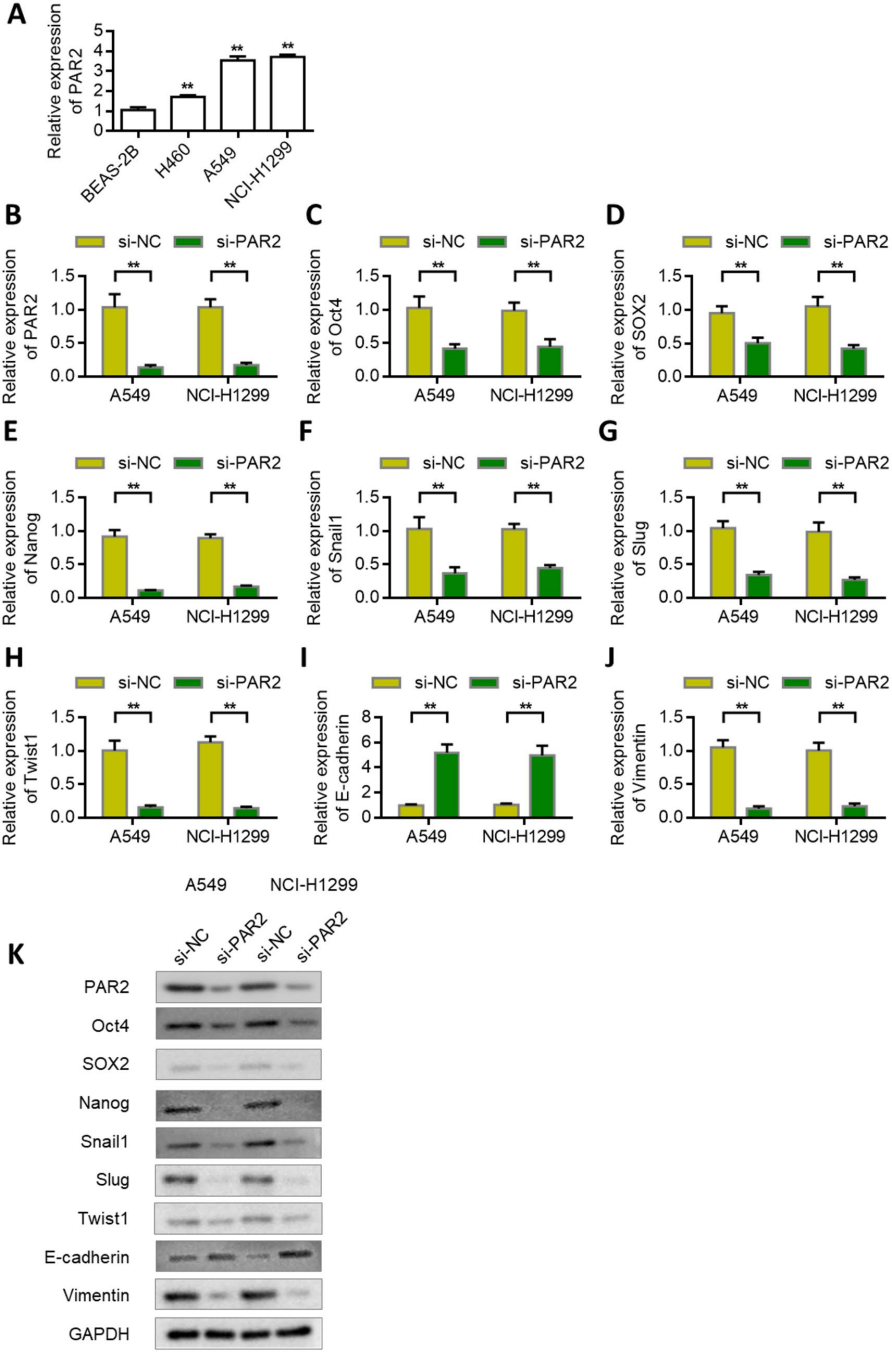
Knockdown of PAR2 inhibits lung cancer cell stemness and EMT. **A** The expression level of PAR2 in NSCLC cell lines and normal bronchial epithelial cell line BEAS-2B was analyzed by RT-qPCR. **B** Verify the efficiency of knocking down PAR2 in A549 and NCI-H1299 cells. **C**-**H** After knocking down PAR2 in A549 and NCI-H1299 cells, the expression of Oct4 (**C**), SOX2 (**D**), Nanog (**E**), Snail (**F**), Slug (**G**), Twist1 (**H**) was detected. **I**-**J**. After knocking down PAR2 in A549 and NCI-H1299 cells, the expression of E-cadherin (**I**) and Vimentin (**J**) was detected by PCR. **K** Western blot analysis of proteins expression after PAR2 silencing in A549 and NCI-H1299 cells. **, P < 0.01. Data are represented as the means ± SD (n = 3)

The expression of PAR2 was higher in A549 and NCI-H1299 cells than in normal lung epithelial cells (Fig. 3A). Therefore, we selected A549 and NCI-H1299 lung cancer cell lines for subsequent studies. To further verify the role of PAR2, we knocked down the expression of PAR2 in A549 and NCI-H1299 cells. Verification results of transfection efficiency showed that siRNA PAR2 could effectively reduce the expression of PAR2. The knockdown efficiency of the siRNA in A549 was 87.2%. The knockdown efficiency of the siRNA in NCI-H1299 was 83.8% (Fig. 3B). We designed two siRNAs (si-PAR2#1, si-PAR2#2). Subsequently, we detected the effects of PAR2 knockdown on the expression levels of stem cell markers OCT4, SOX2, and Nanog. qRT-PCR results showed that PAR2 knockdown decreased the expression levels of OCT4, SOX2, and Nanog (Figs. 3C–E). It also decreased the expression levels of Snail1, Slug, and Twist1 in A549 and NCI-H1299 cells (Figs. 3F–H). Further results showed that PAR2 knockdown upregulated the expression of epithelial marker E-cadherin while decreased the expression of mesenchymal marker vimentin (Figs. 3I, J). The results of western blot were consistent with those of PCR (Fig. 3K). After silencing PAR2, tumor-related proteins expression was inhibited.

### PAR2 knockdown inhibited the proliferation, invasion, and clone formation of lung cancer cells

**Fig. 4:**
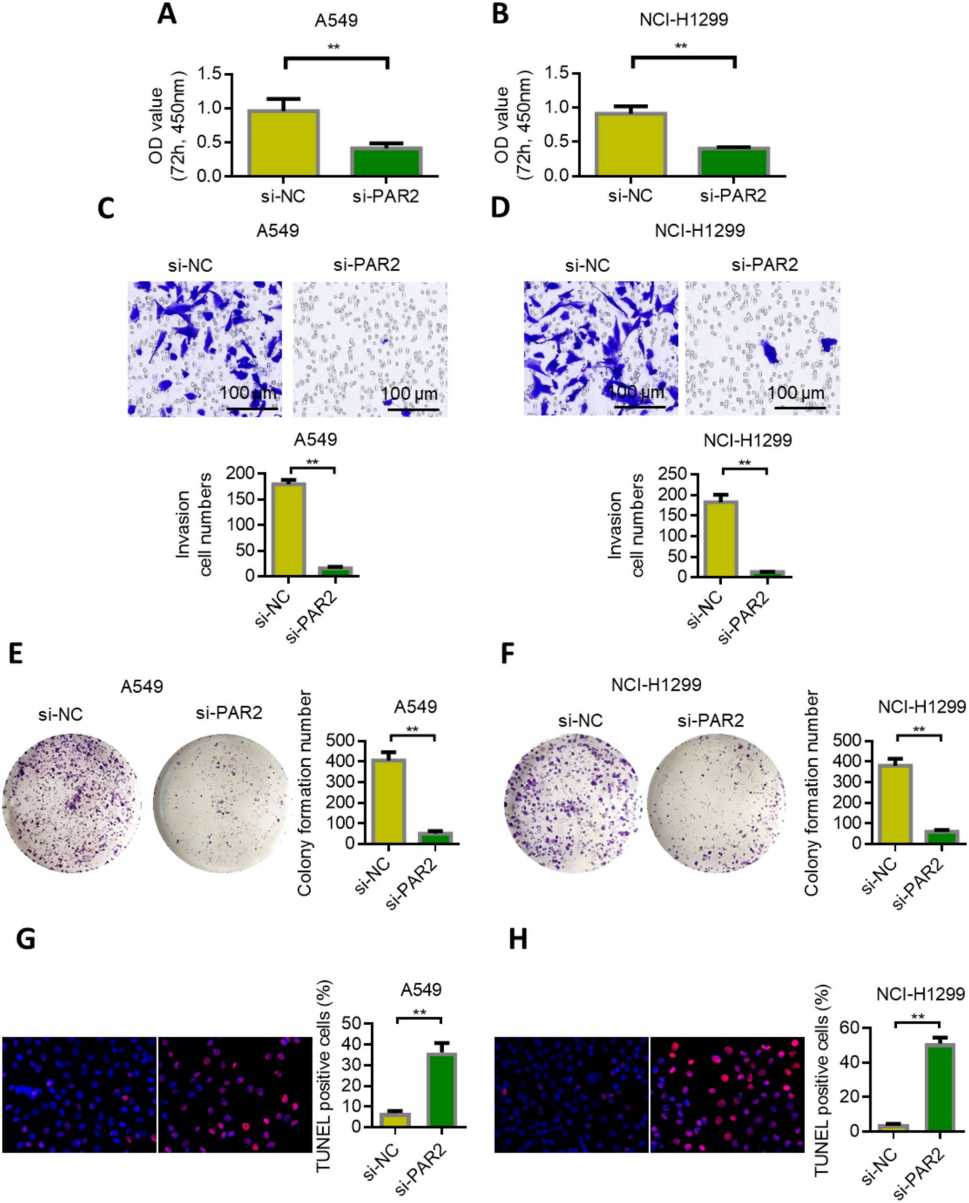
Knockdown of PAR2 inhibits lung cancer proliferation, invasion and clone formation. **A** Knockdown of PAR2 inhibits the proliferation of lung cancer cell A549. **B** Knockdown of PAR2 inhibits the proliferation of lung cancer cell NCI-H1299. **C** Knockdown of PAR2 inhibits the invasion of lung cancer cell A549. D. Knockdown of PAR2 inhibits the invasion of lung cancer cells NCI-H1299. **E** Knockdown of PAR2 inhibits the cloning ability of lung cancer cell A549. **F** Knockdown of PAR2 inhibits the clonogenic ability of lung cancer cell NCI-H1299. **G**-**H** After the silencing of PAR2, the apoptosis rate of A549 and NCI-H1299 cells was up-regulated. **, P < 0.01. Data are represented as the means ± SD (n = 3)

The expression of PAR2 was knocked down in lung cancer A549 and NCI-H1299 cells, and the changes in proliferation, invasion, and clone formation were detected. MTT results showed that the proliferation rate of both PAR2 knockdown cells significantly decreased compared with the control group (Figs. 4A, B). Transwell experiment results showed that the invasion of the siPAR2-transfected cells significantly decreased compared with that of the siNC group (Figs. 4C, D). The results of the clone formation experiment showed that the clone formation of the siPAR2-transfected cells significantly decreased compared with that of the SINC group (Figs. 4E, F). These results indicate that the low expression of PAR2 in lung cancer can inhibit the proliferation, invasion, and clone formation of cells. We further supplemented the effect of silencing PAR2 on apoptosis through TUNEL assay. TUNEL results showed that apoptosis rates of A549 and NCI-H1299 cells were significantly up-regulated after PAR2 silenced (P < 0.01). The experimental results are supplemented in Figs. 4G, H.

### PAR2 knockdown can increase chemotherapeutic sensitivity of gefitinib in lung cancer

**Fig. 5:**
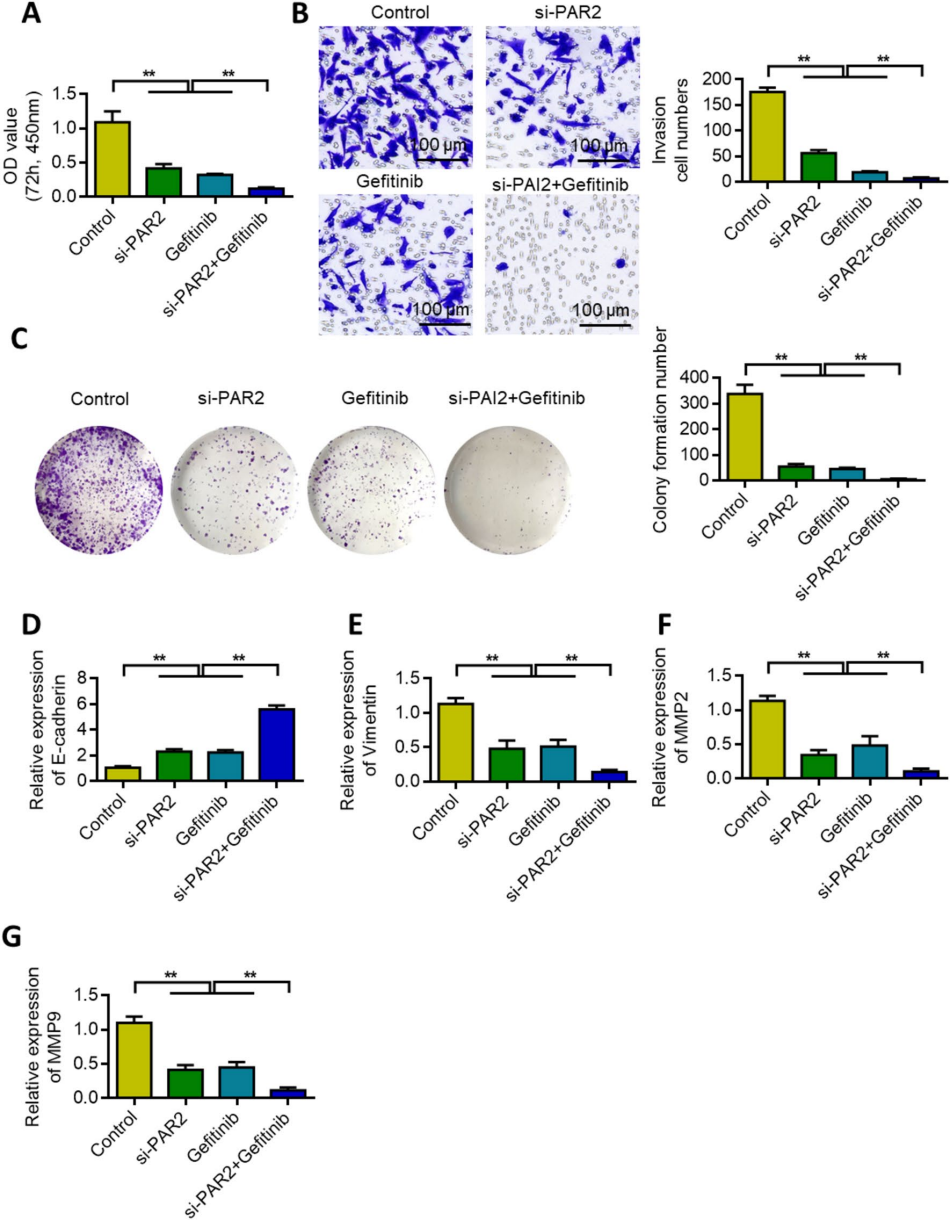
Knockdown of PAR2 can increase the sensitivity of Gefitinib to lung cancer chemotherapy. **A** After different treatments, MTT test detects cell proliferation. **B** After different treatments, cell Transwell detection. **C** After different treatments, the cell clone formation ability test. **D**-**G** qRT-PCR detects changes in E-cadherin (**D**), Vimentin (**E**), MMP2 (**F**) and MMP9 (**G**) expression after different treatments. **, P < 0.01. Data are represented as the means ± SD (n = 3)

MTT assay was used to investigate the effect of PAR2 knockdown and gefitinib alone or in combination on the survival rate of lung cancer cells. Results showed that either PAR2 knockdown alone or gefitinib treatment reduced the survival rate of lung cancer cells. PAR2 knockdown increased further gefitinib inhibition (Fig. 5A). Transwell was used to evaluate the invasiveness of cells. Either PAR2 knockdown alone or gefitinib treatment reduced the invasion of lung cancer cells. PAR2 knockdown further increased the inhibitory effect of gefitinib (Fig. 5B). The inhibitory effect of PAR2 knockdown and gefitinib on the colony formation of NSCLC cells was tested by colony formation assay. Results showed that either PAR2 knockdown alone or gefitinib treatment reduced the ability of lung cancer cells to form clones. PAR2 knockdown further increased the inhibitory effect of gefitinib (Fig. 5C). When PAR2 was knocked down and combined with gefitinib, E-cadherin expression was upregulated (Fig. 5D), but vimentin expression was inhibited (Fig. 5E). PAR2 knockdown alone or treatment with gefitinib decreased MMP2 and MMP9 expression in lung cancer cells. However, PAR2 knockdown further increased the inhibitory effect of gefitinib (Figs. 5F–G).

### Melittin inhibited PAR2 and malignant progression of lung cancer cells

**Fig. 6:**
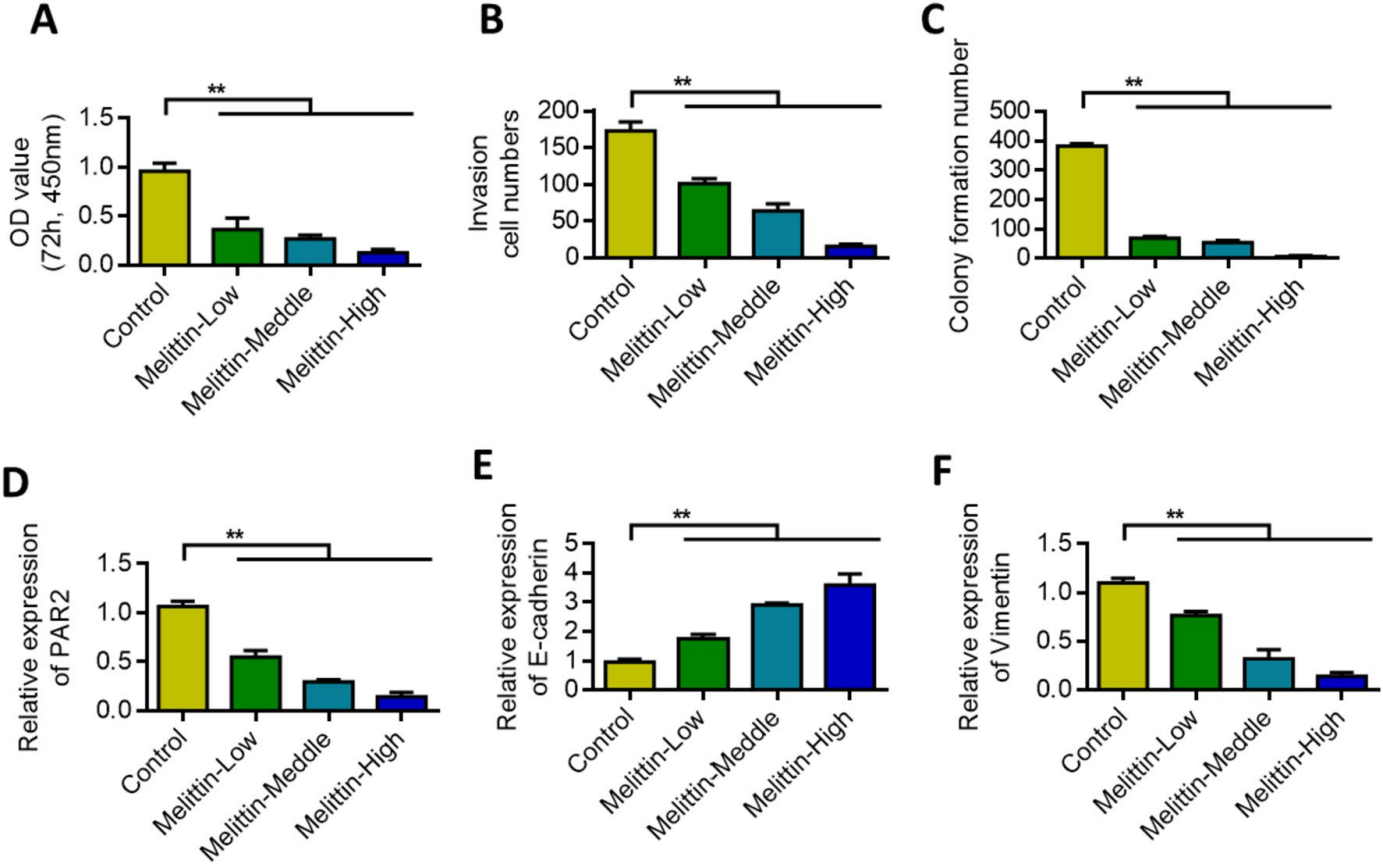
Melittin inhibits PAR2 and inhibits the malignant progression of lung cancer cells. **A** After treatment with different concentrations of Melittin, cell proliferation detection. **B** After treatment with different concentrations of Melittin, the cells are tested by Transwell. **C** After treatment with different concentrations of Melittin, cell clone formation ability detection. **D** qRT-PCR detects the changes in PAR2 expression after different concentrations of Melittin are treated. **E** qRT-PCR detects the changes in E-cadherin expression after different concentrations of Melittin are treated. **F** qRT-PCR detects the changes in Vimentin expression after different concentrations of Melittin are treated. **, P < 0.01. Data are represented as the means ± SD (n = 3)

MTT assay, Transwell assay, and colony formation assay were used to detect the effects of melittin on the proliferation, invasion, and colony formation of lung cancer cells. Results showed that melittin inhibited the proliferation, invasion, and colony formation of lung cancer cells in a dose-dependent manner (Figs. 6A–C). Melittin inhibited the expression of PAR2 in a dose-dependent manner (Fig. 6D). EMT marker detection results showed that melittin could dose-dependently upregulate the expression of E-cadherin and inhibit the expression of vimentin (Figs. 6E, F).

### Melittin increases gefitinib sensitivity to lung cancer chemotherapy by inhibiting PAR2

**Fig. 7:**
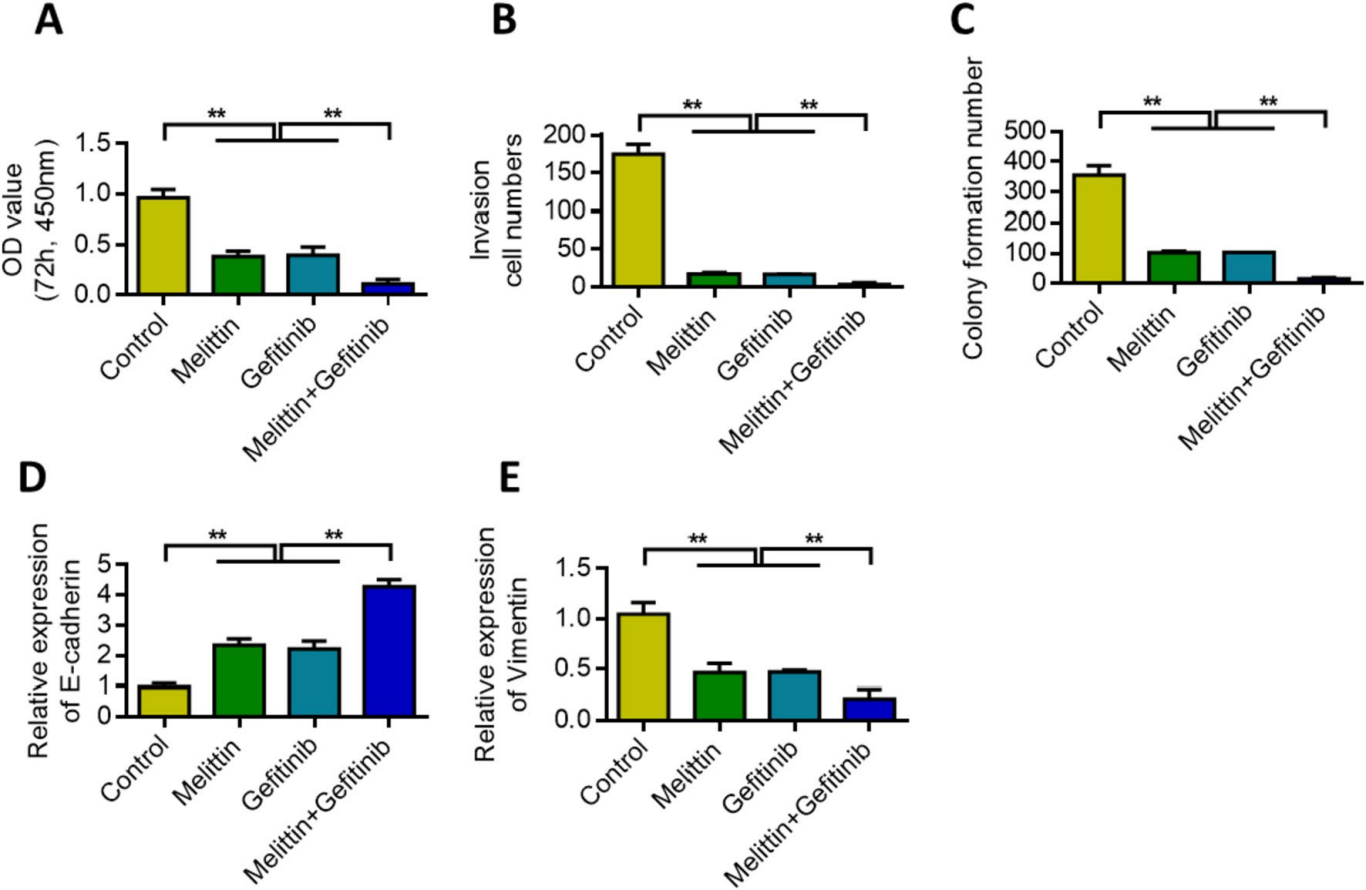
Melittin increases the sensitivity of Gefitinib to lung cancer chemotherapy by inhibiting PAR2. **A** After combined treatment with Melittin and Gefitinib, cell proliferation was detected. **B** After combined treatment with Melittin and Gefitinib, cell Transwell detection. **C** Melittin combined with Gefitinib, the cell clone formation ability was detected. **D** qRT-PCR detects the changes in the expression of E-cadherin after the combined treatment of Melittin and Gefitinib. **E** qRT-PCR detects the changes in Vimentin expression after Melittin and Gefitinib are combined. **, P < 0.01. Data are represented as the means ± SD (n = 3)

MTT assay was used to analyze the effect of melittin and gefitinib on the survival rate of lung cancer cell lines. Results showed that melittin alone or gefitinib treatment reduced the survival rate of lung cancer cells. Melittin increased the inhibitory effect of gefitinib (Fig. 7A). Transwell results showed that melittin alone or gefitinib treatment reduced the invasion of lung cancer cells. Melittin further increased the inhibitory effect of gefitinib (Fig. 7B). Colony formation experiments showed that melittin alone or gefitinib treatment reduced the colony formation of lung cancer cells. Melittin further increased the inhibitory effect of gefitinib (Fig. 7C). When melittin was combined with gefitinib, E-cadherin expression was upregulated (Fig. 7D), but vimentin expression was inhibited (Fig. 7E).

## Discussion

PARs, a family of protease-activated receptors, are G-protein-coupled receptors (GPCR). The PAR family, which has four members, plays an important role in vascular physiology, neural tube closure, hemostasis, and inflammation [29]. PAR1 and PAR2 promote the invasion and metastasis of some type tumor cells by regulating cell migration and angiogenesis and interacting with platelets, fibroblasts cells [30–33]. The regulatory effects of PAR2 on tumor progression are multifaceted. PAR2 activation promotes the release of vascular endothelial growth factor, interleukin-6, and interleukin-8, thereby promoting the formation and invasion of new blood vessels in malignant tumors [34]. PAR2 is also necessary for coagulation factors VII A and PARA to induce the metastasis and invasion of breast cancer cells [35]. In addition, PAR2 activation promotes the reversal of epidermal growth factor (EGF) and the release of TGF-α, induces tumor angiogenesis, and mediates the proliferation of gastric, colon, and esophageal cancer cells [36].

In the present study, the mRNA levels of PAR2 significantly increased in NSCLC tissues compared with paracancerous tissues. The expression of PAR2 and its clinical significance were also studied in this study. Survival analysis showed that patients with high PAR2 expression had shorter overall survival than those with low PAR2 expression. Therefore, PAR2 in lung cancer tissue may be used as a molecular target for the prognosis of lung cancer patients, assisting in the clinical diagnosis and treatment of lung cancer to a certain extent. We further investigated whether or not PAR2 expression could affect the proliferation and invasion of NSCLC cells. We transfected si-PAR2, which could interfere with PAR2 expression, into NSCLC A549 and NCI-H1299 cells to obtain cell lines with low PAR2 expression. MTT assay results showed that the proliferation of PAR2 knockdown cells was significantly reduced compared with the control group. Transwell assay results also confirmed that PAR2 knockdown not only affected the proliferation of NSCLC cells but also significantly reduced their invasion compared with the control group. Moreover, PAR2 knockdown increased the inhibitory effect of gefitinib on lung cancer.

In this study, we examined the effects of melittin at different concentrations on the proliferation, invasion, and clone formation of NSCLC cells A549 and NCI-H1299. Results showed that low-dose melittin can effectively inhibit the proliferation of these cells in a dose-dependent manner. As one of the basic principles of tumor chemotherapy, drug combination aims to achieve synergistic effects, reduce drug dosage, and reduce adverse reactions [37, 38]. In the present study, using the principle of neutral effect, lung cancer cell lines A549 and NCI-H1299 were used as research objects to study the interaction between melittin and gefitinib by inhibiting PAR2.

In the present study, melittin and gefitinib showed significant synergistic effects on lung cancer cell lines. Existing antitumor studies of melittin mostly focused on the inhibition of cell proliferation, induction of apoptosis, or inhibition of angiogenesis, but the underlying molecular mechanism remains to be elucidated. Results of this study revealed that melittin can inhibit the PAR2 pathway in NSCLC cells and thus inhibit EMT. Thus, melittin can be used to achieve better efficacy than gefitinib alone.

There are limitations to the study. Whether Melittin has other targets remains to be further studied. In addition, this study demonstrated the role of PAR2 in lung cancer and the antitumor effect of Melittin only at the cellular level. Melittin has not been proven to have an anti-tumor effect or to increase the anticancer effect of chemotherapy in vivo. The present study also lack of using multiple concentrations of PAR2 and Gefitinib for subsequent studies. In the future, we will consider experiments on drug-resistant lung cancer cell lines to further demonstrate the effect of Melittin on the improvement of gefitinib resistance by inhibiting PAR2.

## Conclusion

PAR2 plays an important role in the occurrence and development of lung cancer. High expression of PAR2 promoted the proliferation, migration, and invasion of lung cancer cells and was significantly correlated with the drug resistance of lung cancer cells. Melittin enhanced the anticancer activity of gefitinib by inhibiting PAR2. The results of this study need to be explored in other types of tumor and validated in future clinical trials.

### Supplementary Information

Below is the link to the electronic supplementary material.

Supplementary file1 Fig. S1. The verification of siRNA PAR2 transfection efficiency in A549 and NCI-H1299 cell lines. The efficiency of transfection was assessed by measuring the expression levels of PAR2. The results demonstrate the successful transfection of siPAR2#1 in both cell lines, as evidenced by the significant decrease in PAR2 expression compared to the control group. **, P < 0.01. ns no significant. Data are represented as the means ± SD (n=3). (DOCX 20 KB)

## Data Availability

The datasets used and analyzed in the current study are available from the corresponding author on reasonable request.

## Abbreviations

PAR2: Protease-activated receptor
EMT: Epithelial mesenchymal transformation;
NSCLC: Non-small cell lung cancer
MMPs: Matrix metallo proteinases
EGFR: Epidermal growth factor receptor
