# Correction to: Inhibition of proteinase-activated receptor 2 (PAR2) decreased the malignant progression of lung cancer cells and increased the sensitivity to chemotherapy

**DOI:** 10.1007/s00280-024-04646-8

**Published:** 2024-03-26

**Authors:** Hongjie Huo, Yu Feng, Qiong Tang

**Affiliations:** Department of Respiration Medicine, Tianjin Union Medical Center, Tianjin, 300121 China

**Correction to: Cancer Chemotherapy and Pharmacology** 10.1007/s00280-023-04630-8

In the original publication, the order of authors have been appeared incorrectly as

Feng Yu^1^ · Hongjie Huo^1^ · Qiong Tang^1^

The correct order should read as below:

Hongjie Huo^1^ · Yu Feng^1^ · Qiong Tang^1^

In addition, we have included funding note to the article as below:

This study was supported by Tianjin Union Medical Center (Grant number 2021YJ020).

The original article has been corrected.

