# Supplementary material for: Inhibition of proteinase-activated receptor 2 (PAR2) decreased the malignant progression of lung cancer cells and increased the sensitivity to chemotherapy
Source: Cancer Chemother Pharmacol. 2024 Jan 4;93(5):397–410. doi: 10.1007/s00280-023-04630-8 (PMC11043148; doi:10.1007/s00280-023-04630-8)
Supplement: Supplementary file 1 — Supplementary file1 Fig. S1. The verification of siRNA PAR2 transfection efficiency in A549 and NCI-H1299 cell lines. The efficiency of transfection was assessed by measuring the expression levels of PAR2. The results demonstrate the successful transfection of siPAR2#1 in both cell lines, as evidenced by the significant decrease in PAR2 expression compared to the control group. **, P < 0.01. ns no significant. Data are represented as the means ± SD (n=3). (DOCX 20 KB) [file 280_2023_4630_MOESM1_ESM.docx]

**Figure S1. The verification of siRNA PAR2 transfection efficiency in A549 and NCI-H1299 cell lines.** The efficiency of transfection was assessed by measuring the expression levels of PAR2. The results demonstrate the successful transfection of siPAR2#1 in both cell lines, as evidenced by the significant decrease in PAR2 expression compared to the control group. **, P < 0.01. ns: no significant. Data are represented as the means ± SD (n=3).
